# Experimental and Computational Investigation of the Target and Mechanisms of *Gelsemium* Alkaloids in the Central Nervous System

**DOI:** 10.3390/ijms26031312

**Published:** 2025-02-04

**Authors:** Yunfan Wang, Zhijiang Yang, Tengxin Huang, Li Pan, Junjie Ding, Zhaoying Liu

**Affiliations:** 1 College of Veterinary Medicine, Hunan Agricultural University, Changsha 410128, China; 18669697363@163.com; 2 State Key Laboratory of NBC Protection for Civilian, Beijing 102205, China; yzjkid9@gmail.com (Z.Y.); h1064482785@163.com (T.H.); bk6180b@163.com (L.P.)

**Keywords:** *Gelsemium* alkaloids, GABAA receptor, glycine receptor, electrophysiology, molecular dynamics simulation

## Abstract

*Gelsemium* has a long history of medicinal use but is also a poisonous plant. Some low-toxicity alkaloids in *Gelsemium* exhibit anxiolytic, anti-inflammatory, analgesic, and other pharmacological effects; however, certain alkaloids in *Gelsemium* are highly toxic. Nevertheless, the molecular targets underlying the biological effects of *Gelsemium* alkaloids remain poorly understood. We employed electrophysiological techniques and molecular modeling to examine the modulatory effects of *Gelsemium* alkaloids on inhibitory neurotransmitter receptors, as well as to elucidate the mechanisms underlying their molecular interactions. Our findings indicate that low-toxicity alkaloids primarily exert their pharmacological effects through actions on glycine receptors, with the binding site located at the orthosteric site between two α-subunits. Both highly toxic and low-toxicity alkaloids target GABAA receptors, using the β+/α− interface transmembrane structural domains as common binding sites. These results identify the targets through which *Gelsemium* alkaloids affect the central nervous system and predict the binding modes and key amino acids involved from a computational modeling perspective. However, further experimental validation through mutational studies is necessary to strengthen these findings.

## 1. Introduction

*Gelsemium* is a flowering plant belonging to the family Loganiaceae that is widely distributed in central and southeastern China. It has a long history of medicinal use in folk medicine [1,2,3,4], and its main active components are alkaloids. To date, over 100 alkaloids belonging to six distinct classes have been isolated and characterized from *Gelsemium*, including gelsedine-, gelsemine-, koumine-, humantenine-, yohimbane-, and sarpagine-type alkaloids. Among these, gelsemine and koumine are recognized as the main active constituents [5,6]. Modern pharmacological studies have shown that these alkaloids exert various pharmacological effects, including anxiolytic [7,8,9], anti-inflammatory [10,11,12], analgesic [13], immunomodulatory [14,15], and antitumor effects [16,17], demonstrating strong potential for medicinal development. However, *Gelsemium* is highly toxic, and there are numerous reports of poisoning or even death due to accidental ingestion, which severely limits its clinical development and utilization. The gelsedine- and humantenine-type alkaloids are identified as the primary toxic components of *Gelsemium*, with the LD_50_ of these two alkaloid types in mice injected intraperitoneally typically being less than 1 mg·kg^−1^ [18,19,20]. Consequently, understanding the regulation and mechanisms of *Gelsemium*’s toxicity is essential to advance its safe use and therapeutic development. *Gelsemium* poisoning is associated with a range of neurotoxic symptoms, including convulsions, chest tightness, and respiratory distress [21,22]. While the toxic effects of *Gelsemium* alkaloids may be related to the modulation of neuronal activity involved in controlling such functions, the molecular targets underlying the biological effects of *Gelsemium* alkaloids in the central nervous system (CNS) remain unclear.

Gamma-aminobutyric acid receptor receptors (GABAARs) and glycine receptors (GlyRs) are ligand-gated ion channels responsible for transmitting inhibitory signals to the CNS [23,24]. Notably, the analgesic mechanisms of koumine and gelsemine may be related to GlyRs in the brain [25]. For example, electrophysiological studies have shown that koumine, gelsemine, and gelsevirine modulate GlyRs. Moreover, amino acid mutagenesis identified F63 of the α-subunit as a key residue in the interaction of these three alkaloids with GlyRs. However, this study did not investigate the interactions of the alkaloids with GlyRs or the contributions of specific amino acids to binding from a molecular modeling perspective [26,27].

Yang et al. extracted 14-(R)-hydroxy-gelsenicine, a highly toxic *Gelsemium* alkaloid, from honey and demonstrated that it significantly prolongs the action potential of the GABAAR. The use of flumazenil, a selective antagonist of the GABAAR, effectively restored action potentials and improved survival in animal models [28]. Similar findings were reported by Li et al., who noted that flumazenil, a GABAAR inhibitor, reversed the channel opening induced by the prolonged action of gelsenicine [29]. This suggests that gelsenicine is a negative modulator of the GABAAR, albeit with a weaker binding affinity than flumazenil. Electrophysiological experiments have shown that gelsenicine significantly reduces the frequency of GABAergic synaptic currents in cortical neurons and inhibits currents generated by GABA agonists, suggesting that gelsenicine may be a negative modulator of GABAARs [30]. However, the mechanisms by which *Gelsemium* alkaloids interact with GABAARs remain unclear.

In this study, we aimed to comprehensively investigate the modulatory effects of five typical *Gelsemium* alkaloids (koumine, gelsemine, gelsevirine, gelsenicine, and humantenirine) on the currents of GABAA, glycine, and acetylcholine ion channels. We also investigated the molecular interactions between gelsemine, gelsenicine, and GABAARs and GlyRs. Our study elucidated the differences in the action targets between low-toxicity and high-toxicity alkaloids, thereby enhancing the understanding of the pharmacological and toxicological properties of *Gelsemium* alkaloids, which supports their potential as clinical drugs.

## 2. Results

### 2.1. Target Confirmation of Gelsemium Alkaloids

We examined the current-modulating effects of five *Gelsemium* alkaloids (koumine, gelsemine, gelsevirine, gelsenicine, and humantenirine) on GABAA, glycine, and acetylcholine ion channels. Single-concentration experiments indicated that the alkaloids did not significantly regulate acetylcholine ion channel currents. Alkaloids exhibited low activity for GABAARs. In contrast, for GlyRs, koumine, gelsemine, and gelsevirine demonstrated strong activity. No significant change in the amplitude of current regulation was observed when comparing homologous α1 Gly receptors with heterologous α1β Gly receptors, suggesting that the α-type subunit plays a major role in Gly receptor binding to *Gelsemium* alkaloids (Table 1).

**Figure 1:**
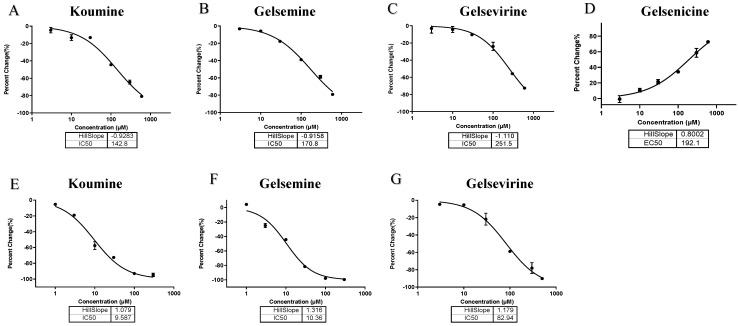
Concentration-response curves of *Gelsemium* alkaloids with α1 GlyR and α1β2γ2 GABAAR, with the horizontal axis representing the concentration and the vertical axis representing the peak current of percentage change. (**A**–**D**) Concentration response curves (0.01–300 µM) of alkaloids on homomeric α1 GlyR. (**E**–**G**) Concentration response curves (0.01–300 µM) of alkaloids on homomeric α1β2γ2 GABAAR. The horizontal coordinate represents the alkaloid concentration (μm) and the vertical coordinate represents the percentage of channel opening, with 6 concentrations per group and 4–5 repetitions per concentration.

Based on the above results, we selected the alkaloids that induced greater than 50% change in channel currents at 300 µM to further investigate their multi-concentration effects on targets (Table 2). The results of the study showed that koumine, gelsemine, and gelsevirine showed IC_50_ values of 142.8 µM, 170.8 µM, and 251.5 µM for the GABAAR, respectively. Gelsenicine increased the current amplitude with an EC_50_ value of 192.1 µM. For GlyRs, koumine, gelsemine, gelsevirine produced a larger current reduction with IC_50_ values of 9.587 µM, 10.36 µM, and 82.94 µM, respectively (Figure 1). Research indicates that the EC_50_ value of diazepam for positive allosteric modulation of GABAAR is approximately 5 nM, while the IC_50_ value of strychnine for GlyR is around 180 nM [31,32]. As a natural product derived from plants, the modulatory activity of *Gelsemium* alkaloids is significantly weaker than that of these classical modulators. Nevertheless, our study primarily focuses on identifying the targets of *Gelsemium* alkaloids, specifically that the main targets of koumine, gelsemine, and gelsevirine are GlyR, while GABAAR serves as a common target for *Gelsemium* alkaloids, despite exhibiting relatively weak modulatory activity.

### 2.2. Target Site Analysis of Gelsemium Alkaloids

**Figure 2:**
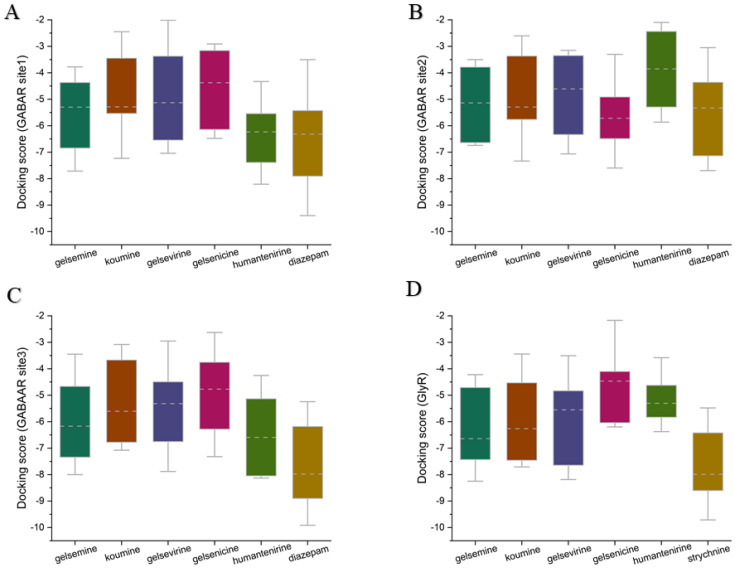
The box plots of molecular docking scores for five *Gelsemium* alkaloids, along with known modulators and their binding sites on GlyR and GABAAR. (**A**) GABAAR Site 1. (**B**) GABAAR Site 2. (**C**) GABAAR Site 3. (**D**) GlyR orthosteric site. The box plots display medians (indicated by the dotted line) and interquartile ranges (25th to 75th percentiles, represented by the box borders). The whiskers extend to the maximum and minimum docking score values. A maximum of 30 binding conformations was retained for each system.

To elucidate the mechanism of action of *Gelsemium* alkaloids on the two ion channels, the two most representative alkaloids, gelsemine (a typical gelsemine-type alkaloid) and gelsenicine (a typical gelsedine-type alkaloid), were selected for this study. Molecular docking and molecular dynamics simulation techniques were used to explore the binding modes and mechanisms of action of these alkaloids on GlyRs and GABAARs. Based on previous studies and experimental data, the extracellular structural domain of the α1 GlyR was identified as a potential target site for *Gelsemium* alkaloids [26]. In addition to the classical benzodiazepine site located in the extracellular structural domain at the α+/γ interface, the GABAAR possesses two additional active sites in the transmembrane structural domain: one at the β+/α interface and another at the γ+/β interface. As no studies have yet explored the site of action of *Gelsemium* alkaloids on the GABAAR, three binding states were identified: the extracellular structural domain of the α/γ interface, the classical benzodiazepine site at the α+/γ interface [33], and the transmembrane structural domain at the γ/β interface, labeled as Sites 1–3, respectively. To assess the reliability of the docking method used in this study, we reproduced the docking of the crystal structures from the PDB database. The root mean square deviation (RMSD) of the re-docked conformations from the complexes in the PDB was less than 1.5 Å (Supplementary Table S1), suggesting that the docking method employed in this study is very reliable and that the docking system is stable. Subsequently, the structures of gelsemine and gelsenicine complexes bound to the GABAAR and GlyR were constructed. Docking score values represent the predicted binding affinities (in kcal/mol), reflecting the molecule’s capacity to interact with a defined binding site. A docking score was computed for each ligand-receptor interaction, and the most negative value indicates a more favorable binding energy, which suggests a more stable ligand-receptor complex. To provide a broader view of potential ligand-receptor interactions, we utilized box plots to describe the complete docking score dataset (Figure 2). At the three binding sites of GABAAR, the binding scores of the five *Gelsemium* alkaloids showed no significant differences, implying that there is no difference in the binding stability of the five alkaloids to GABAAR. On the other hand, the median docking scores of koumine, gelsemine, and gelsevirine on GlyR were significantly higher than those of gelsenicine and humantenirine, suggesting that these compounds have more stable binding. In addition, the known regulators diazepam and strychnine both had better binding scores than *Gelsemium* alkaloids, suggesting that *Gelsemium* alkaloids may have a weaker affinity for GABAAR and GlyR than diazepam and strychnine. Next, the binding conformations with the best binding scores in each group of systems were selected for molecular dynamics simulation studies.

**Figure 3:**
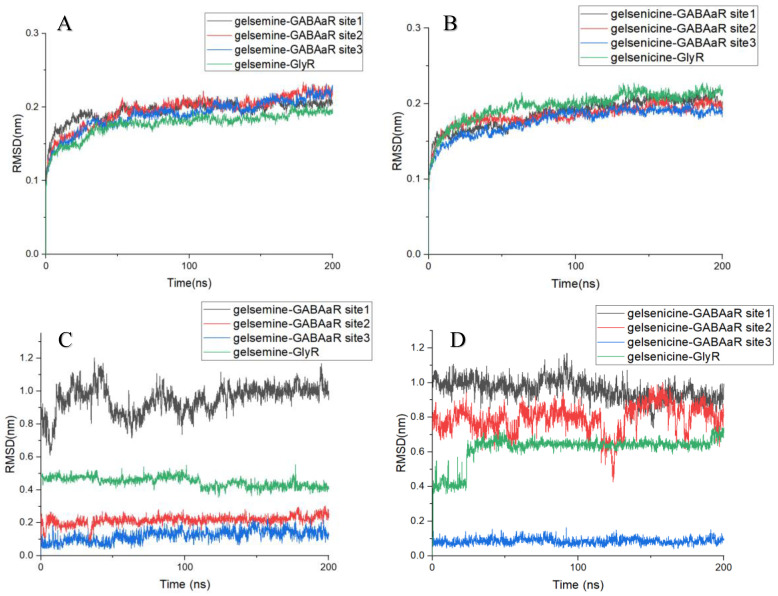
(**A**,**B**) RMSD of the protein backbone (**C**,**D**). RMSD between protein and ligand in complex. The *x*-axis represents simulation time (in ns), while the *y*-axis denotes RMSD (in nm).

The RMSD is a key criterion for assessing the conformational stability of proteins and ligands in molecular dynamics simulations [34]. The RMSD analysis of the protein backbone for each system showed that the RMSD of each system increased gradually within 50 ns and reached equilibrium with an average RMSD of approximately 0.2 nm. All the simulated trajectories equilibrated within 200 ns, and the simulation time was sufficient for further analysis. Comparing the RMSD between the ligand and receptor in each complex, the conformational stability of alkaloid binding at the active site during the molecular dynamics simulation can be assessed. In the four gelsemine systems, the RMSD of gelsemine-GABAAR Site 1 exhibited significant fluctuations from the beginning of the simulation, eventually stabilizing at around 1.0 nm, and the RMSD of gelsemine-GABAAR Site 2 remained stable at approximately 0.5 nm, indicating that gelsemine shifted in these two active sites during binding. In contrast, for gelsemine-GABAAR Site 3, gelsemine binding was stable at both GABAAR Site 3 and the GlyR binding site, with stable RMSD of approximately 0.2 nm and 0.15 nm, respectively. This suggests that gelsemine consistently occupied these two pockets. Meanwhile, gelsenicine exhibited stable binding only at GABAAR Site 3, with an RMSD of approximately 0.15 nm. In contrast, the RMSD at the other three sites exhibited significant fluctuations, indicating that gelsenicine did not bind well at these three sites (Figure 3).

**Figure 4:**
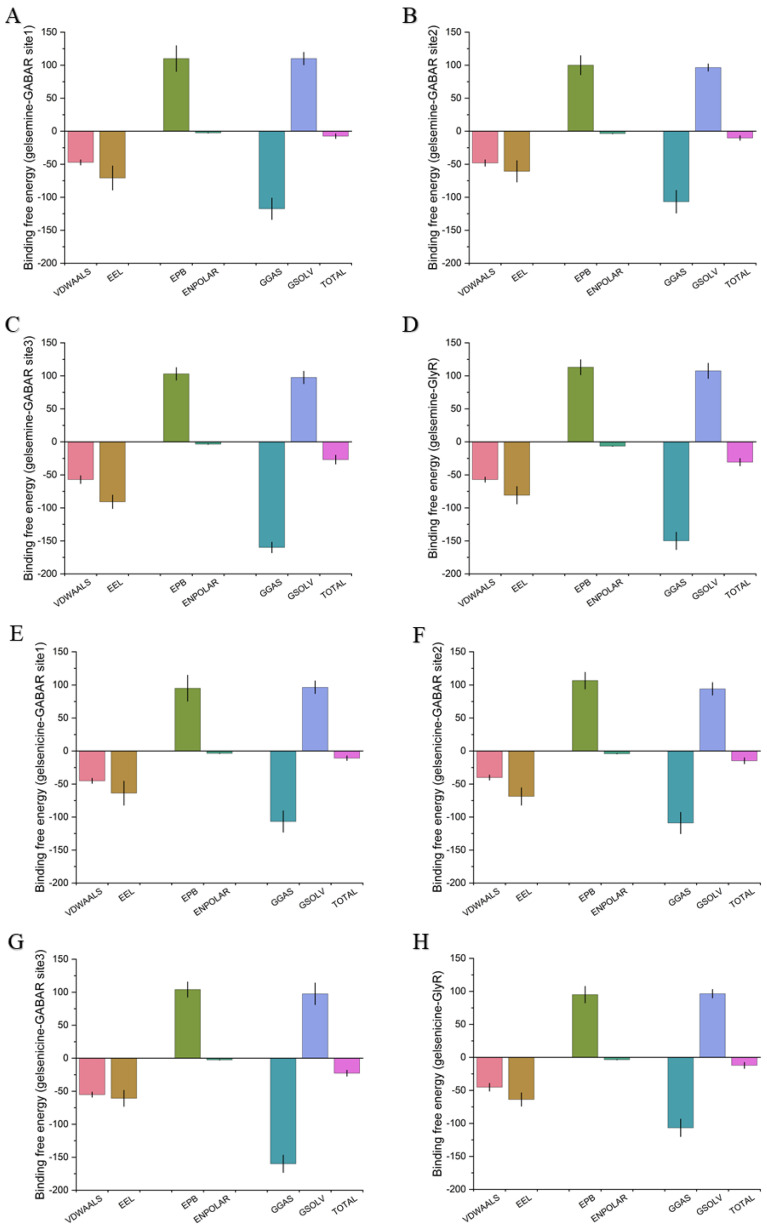
Binding free energy of gelsemine-GABAAR Site 1 (**A**), gelsemine-GABAAR Site 2 (**B**), gelsemine-GABAAR Site 3 (**C**), gelsemine-GlyR (**D**), gelsenicine-GABAAR Site 1 (**E**), gelsenicine-GABAAR Site 2 (**F**), gelsenicine-GABAAR Site 3 (**G**), and gelsenicine-GlyR (**H**). The *x*-axis displays the total binding free energy along with six components of the energy decomposition. TOTAL = GGAS + GSOLV = (VDWAALS + EEL) + (EPB + ENPOLAR). The *y*-axis represents the binding free energy (in kcal/mol).

The binding free energy results corroborated this phenomenon, showing that the order of favorable binding interactions was gelsemine-GlyR > gelsemine-GABAAR Site 3 > gelsemine-GABAAR Site 2 > gelsemine-GABAAR. Gelsenicine demonstrated binding strength only at GABAAR Site 3, consistent with experimental findings (Table 3). These results suggest that the GlyR is the most likely active site for the action of gelsemine, and gelsenicine has a more limited effect on the GABAAR. Based on this, we further analyzed the simulation process of gelsemine-GABAAR Site 3, gelsemine-GlyR, and gelsenicine-GABAAR Site 3 (Figure 4).

### 2.3. Analysis of the Binding Mode of Gelsemium Alkaloids to Targets

**Figure 5:**
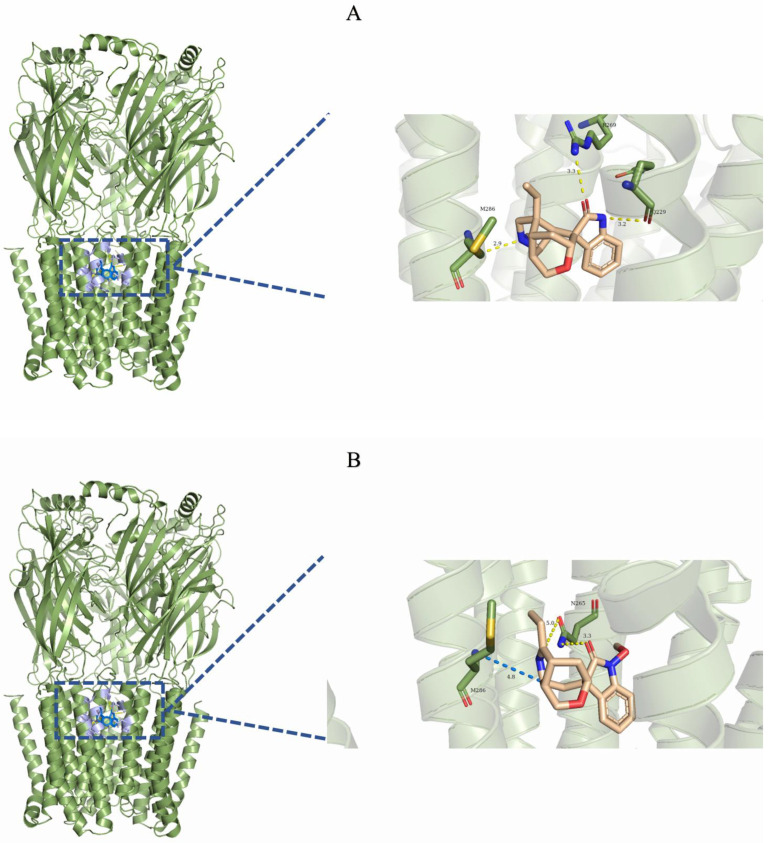

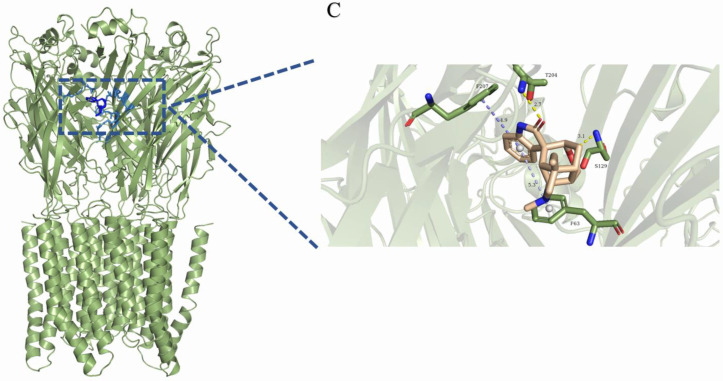
Three-dimensional binding mode of the final frame from the molecular dynamics simulation. (**A**) Gelsemine-GABAAR Site 3. (**B**) Gelsenicine-GABAAR Site 3. (**C**) Gelsemine-GlyR. The yellow dotted line indicates the hydrogen bonding interaction. The light-blue dotted line denotes the pi-pi stacking interaction. The blue dotted line denotes the alkyl stacking interaction.

**Figure 6:**
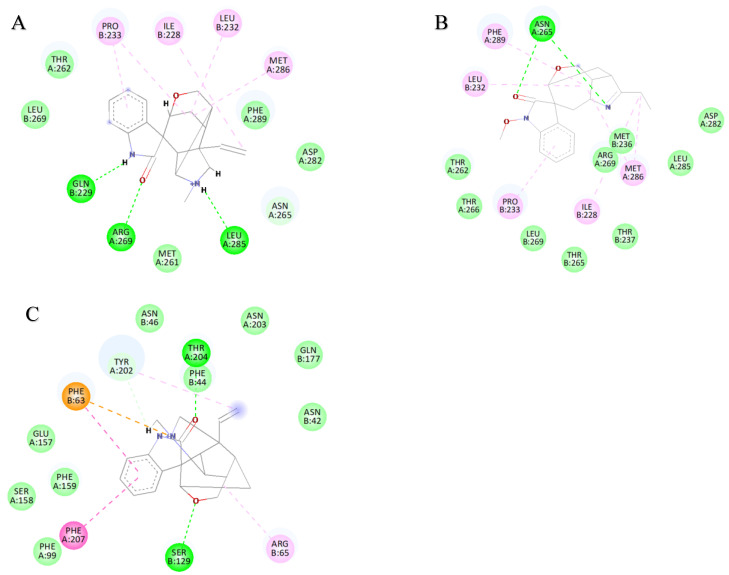
Two-dimensional binding models. (**A**) Gelsemine-GABAAR Site 3. (**B**) Gelsenicine-GABAAR Site 3. (**C**) Gelsemine-GlyR. The purple dotted line denotes the pi-sigma interaction, the dark pink dotted line denotes the pi-pi stacking interaction, the light pink dotted line represents the alkyl interaction, and the green dotted line indicates the hydrogen bonding interaction.

**Figure 7:**
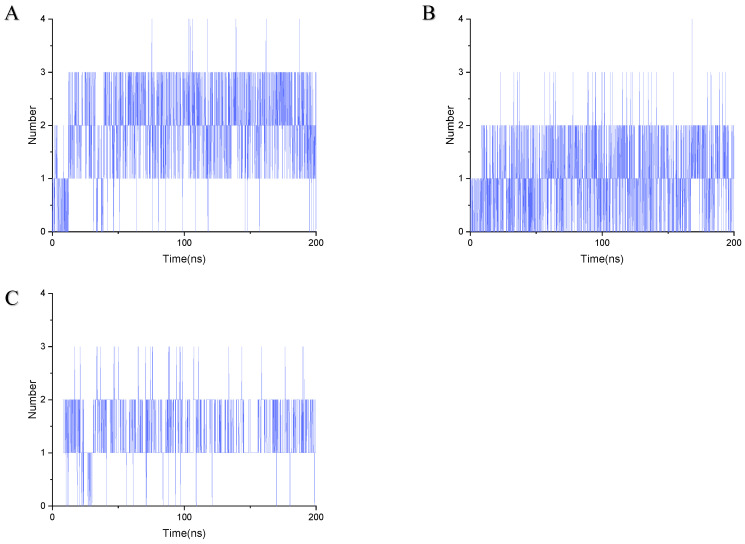
Number of hydrogen bonds formed during 200 ns simulation. (**A**) Gelsemine-GABAAR Site 3. (**B**) Gelsenicine-GABAAR Site 3. (**C**) Gelsemine-GlyR.

**Figure 8:**
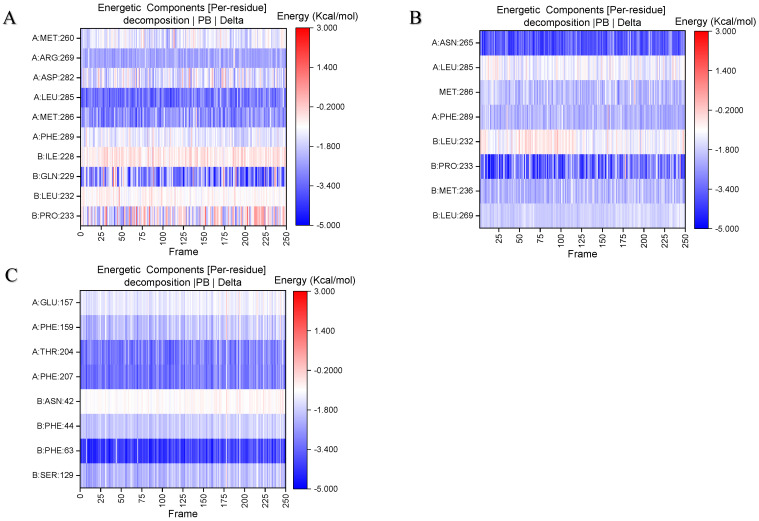
Energy contributions of amino acids residues within 4 Å of the binding site during molecular dynamics simulation. A color shift towards blue indicates a greater energy contribution from the amino acid residues. (**A**) Gelsemine-GABAAR Site 3. (**B**) Gelsenicine-GABAAR Site 3. (**C**) Gelsemine-GlyR.

Stable binding strength depends on the non-bonding interactions between the ligand and receptor. By analyzing the final frames of the simulated trajectories for gelsemine-GABAAR Site 3, gelsemine-GlyR, and gelsenicine-GABAAR Site 3, it was found that hydrogen bonding and hydrophobic interactions were the primary modes of interaction between the *Gelsemium* alkaloids and the aforementioned active sites (Figure 5). Gelsemine formed hydrogen bonds with Gln229 of the α1 subunit and with Arg269 and Leu285 of the β2 subunit of the GABAAR, as well as hydrophobic interactions with Leu232 and Pro233 of the α1 subunit, and Met286 of the β2 subunit. In contrast, gelsenicine established a hydrogen bond with Asn265 of the β2 subunit of the GABAAR, and hydrophobic interactions with Leu232 and Pro233 of the β2 subunit, along with several residues of the α1 subunit, including Leu232, Pro233, Ile228, Phe289, and Met286. Gelsemine formed a π-π stacking interaction with PHE63 and PHE207 on the α1 subunit of the GlyR, as well as an additional π-cation interaction with PHE63. It established hydrogen bonds with both THR204 and SER129, and interacted with ARG65 and TYR202 to create an alkyl hydrophobic interaction (Figure 6). The hydrogen bond formation was individually identified and recorded for the simulated trajectories. The results showed that gelsenicine and GABAARs maintained 0–2 hydrogen bonds, whereas the gelsenicine and GABAARs maintained 2–3 hydrogen bonds. In addition, the gelsemine and Gly receptors exhibited 1–2 hydrogen bonds over the same time period throughout the simulation (Figure 7). To further investigate the mechanism of interaction between the alkaloid and GlyRs and GABAARs, the energy contribution of amino acid residues within 4 Å of the alkaloid in the complex was analyzed using MM-PBSA decomposition. The results shown in Figure 6 indicate that most of the amino acids have negative energy values, suggesting that they are involved in the binding of *Gelsemium* alkaloids to the GABAAR. In terms of energy contribution, Arg269, Leu285, Met286, and Phe289 on the α-chain and Gln229 and Pro233 on the β-chain contributed the most to the binding energy of gelsemine. For gelsenicine, ASN265, Met286 and Phe289 on the α-chain and Pro233 and Met236 on the β-chain contributed the most to the binding energy. PHE63, TYR204, and PHE207 on the α1 subunit of the GlyR play an important role in the binding of gelsemine (Figure 8).

## 3. Discussion

Using electrophysiological techniques combined with molecular simulations, we investigated the molecular determinants of the modulation of inhibitory receptor function by *Gelsemium* alkaloids. Our study revealed different targets and mechanisms of action for low- and high-toxicity alkaloids. In a previous study, three low-toxicity alkaloids, koumine, gelsemine, and gelsevirine, were identified as the primary components contributing to the therapeutic effects of *Gelsemium* on psychiatric disorders, and GlyR may serve as the target of their pharmacological action [35,36]. Our study demonstrated that koumine, gelsemine, and gelsevirine reduced the peak glycine ion channel current, demonstrating inhibitory activity on glycine ion channels. Mutation of F63 on the α-subunit to alanine results in the loss of gelsemine’s ability to regulate GlyR, which is corroborated by our binding free energy decomposition calculation, and our studies show that F63 plays the most important role in the binding of gelsemine to GlyR [26]. Additionally, the electrophysiological results indicated that the three weakly inhibited the currents generated by GABA agonism at subsaturated concentrations. Natural GABAARs expressed in cultured cerebral cortical neurons can be inhibited by gelsemine, which causes a significant reduction in the frequency of spontaneous currents at the GABAergic and glutamatergic synapses. We explored the binding site of gelsemine on GABAAR and found that the binding free energy is highest when it interacts with the active site located in the transmembrane region of the β+/α− interface, and this position is also a potential site for the action of neurosteroids and benzodiazepine drugs [37,38]. The results of the free energy decomposition calculations indicate that amino acids PHE289 and MET286 in the β-subunit contribute the most energy during the binding process, and diazepam forms alkyl interactions with these two amino acids during binding to GABAAR (Supplementary Figure S1). However, their role as key amino acids for binding requires validation through site-directed mutagenesis experiments.

Our results indicate that 300 µM of gelsenicine and humantenirine had a minimal effect on GlyR currents, consistent with previous reports suggesting that GlyRs may not be toxic targets for this alkaloid group. Unlike koumine, gelsemine, and gelsevirine, gelsenicine lacks a positively charged nitrogen group, which is crucial for alkaloid binding to GlyRs. The absence of this nitrogen group prevents the formation of a π-cation interaction with the key amino acid PHE63, which may account for gelsenicine’s inability to modulate GlyR sensitivity. This is consistent with previous findings that gelsenicine does not alleviate anxiety by acting on GlyR [35]. Our team has consistently demonstrated that gelsenicine prolongs the interaction time of GABA with GABAAR and the opening time of chloride ion channels, thus exerting an effect on CNS neurons [39]. This finding was further corroborated in the present study, where gelsenicine regulated GABAAR ion channel currents with an EC_50_ value of 192.1 μM. Using molecular modeling methods, we found that although gelsenicine regulates GABAARs in an opposite manner to gelsemine, the active binding site appears to be the same for both. Notably, the LD_50_ value of gelsenicine/humantenidine is hundreds of times higher than that of gelsemine/koumine [40]. Despite the complexity of the toxicity of these alkaloids in vivo, our study indicated a correlation between alkaloid toxicity and inhibitory neuroreceptor activity. Low-toxicity alkaloids primarily target the GlyR, whereas high-toxicity alkaloids do not affect GlyRs. GABAARs were a common target for all five alkaloids in our study. However, the modulation of GABAARs by low- and high-toxicity alkaloids was diametrically opposed. These results demonstrate that the differential modulation of inhibitory neuroreceptors by *Gelsemium* alkaloids is at least partially responsible for their widely varying toxicities. Although this study identified the GABAAR as a target of action of highly toxic alkaloids, its activity was weak. Given the significant toxicity of these alkaloids, it is reasonable to suspect that there are other important targets involved in their toxicity processes. Preliminary proteomic data from our group have indicated future research directions [41,42], focusing on mapping the interactions of highly toxic alkaloids like gelsenicine with various enzymes in the Gly and GABA pathways and with membrane ion channel receptors.

## 4. Materials and Methods

### 4.1. Chemicals

Gelsemine, koumine, gelsevirine, gelsenicine, and humantenmine (>99% purity) were purchased from Tao Shu (Shanghai, China). All other chemicals were procured from CORNING (Corning, NY, USA), Avantor (Radnor Township, PA, USA), Sigma-Aldrich (St. Louis, MO, USA), or General Reagents.

### 4.2. Cell Culture, Plasmids, and Transfection

HEK293 cells were obtained from Beijing Aisiyipu Biotechnology Co. Cells were cultured in Dulbecco’s Modified Eagle Medium containing 10% fetal bovine serum, 800 µg/mL G418, 200 µg/mL hygromycin B, and 100 µg/mL Zeocin at 37 °C and 5% CO_2_. Cells were transfected with plasmids encoding the following proteins: (i). nAChRα7 (CHRNA7, NM_000746; RIC3, NM_024557), (ii) GABA-α1 (NM_000806), GABA-β2 (NM_021911), and GABA-γ2 (NM_198904), and (iii) GlyR α1 (NM_000171) and GlyR β (NM_000824). To obtain β-containing GlyRs, an α/β transfection ratio of 1:5 was used; for γ2-containing GABAARs, the ratio was 1:2:5 of α/β/γ2.

### 4.3. Electrophysiology

For whole-cell recording, the intracellular contained 140 mM NaCl, 3.5 mM KCl, 1 mM MgCl_2_•6H_2_O, 2 mM CaCl_2_•2H_2_O, 10 mM D-glucose, 10 mM HEPES, and 1.25 mM NaH_2_PO_4_•2H_2_O, (pH 7.4). The extracellular solution contained 50 mM CsCl, 10 mM NaCl, 10 mM HEPES, 60 mM CsF, and 20 mM EGTA (pH 7.2). Briefly, during the diaphragm clamp operation, the glass capillary was drawn into a recording electrode using a microelectrode puller. Subsequently, the electrode filled with intracellular fluid was loaded into the microelectrode holder, and the cell-lined coverslip was placed in the recording bath under an inverted microscope, followed by manipulation of the microelectrode manipulator to immerse the electrode into the extracellular fluid under an inverted microscope. Electrode resistance (Rpip) was recorded, and the electrode was gently positioned on the cell surface with negative pressure applied to establish a GΩ-level high-resistance seal. Fast capacitance compensation was performed, followed by additional suction to breach the cell membrane and form a whole-cell recording. Finally, slow capacitance compensation was performed, and experimental parameters such as series resistance (Rs) were recorded. Leakage compensation was not provided. Whole-cell patch-clamp recordings were performed at −70 mV using HEKA EPC-10 (HEKA Elektronik GmbH, Reutlingen, Germany) amplifiers. Data acquisition was performed using the HEKA PatchMaster. We aimed to study the allosteric regulation of GABAAR and GlyR by *Gelsemium* alkaloids, so the effects of the alkaloids on Gly- or GABA-evoked currents were determined via co-application of subsaturated agonist concentrations (EC_10_ and EC_50_) together with the alkaloid. The EC_50_ for α1 GlyR was 30 µM, whereas the EC_10_ for α1β2γ2 GABAAR was 3.0 µM. The choice of specific agonist concentration was determined based on previous studies [26,43]. We first examined the effects of each *Gelsemium* alkaloid at a concentration of 300 µM on channel currents. The peak receptor currents for subsaturated concentrations of specific agonists and the peak receptor currents after administration of each drug were recorded, and the modulation percentage was calculated for each drug. The modulation percentage was calculated using Equation (1):

Percentage change = ((*I_alkaloid_* − *I_agonist_*)/*I_agonist_*), (1)

where *I_alkaloid_* denotes the current in the presence of a given concentration of alkaloid, and *I_agonist_* denotes the amplitude of the control glycine or GABA current elicited by the activation of a given receptor. To construct the concentration-response curve, six graded concentrations (1 µM, 3 µM, 10 µM, 30 µM, 100 µM, and 300 µM) of each *Gelsemium* alkaloid were tested. The concentration-response curve parameters for alkaloid inhibition were obtained from the curve of the normalized concentration-response data points fitted using Equation (2):

*I_agonist_* = *I*max (agonist)nH/((agonist)nH + (*EC_50_*) nH), (2)

where *I_agonist_* is the current in the presence of a given subsaturated concentration of GABA, and EC_50_ is the concentration required for the half-maximal response. Each drug was repeated four times per concentration, and the data were expressed as the mean ± SE All electrophysiological tests performed at 20 °C.

### 4.4. Molecular Docking and Molecular Dynamics Simulations

GABAAR (α1β2γ2) and GlyR (α1β) structures were obtained from the Protein Data Bank (PDB) database (PDB ID: 6 × 3X, 7TU9). The structures of gelsemine (CAS: 509–15–9) and gelsenicine (CAS: 82354–38–9) were obtained from PubChem. Proteins and small molecules were hydrogenated, structurally optimized, energetically optimized, and protonated at pH 7 ± 0.2 using Schrödinger software (2021.4). Using the ligand-docking module, gelsemine and gelsenicine were docked at protein active sites with ligand flexibility, and the precision was set to XP (extra precision). To validate the docking method, the same approach was used to redock the original ligands from the crystal structures (diazepam in GABAAR and strychnine in GlyR). After docking, the structure with the optimal binding score was used for molecular dynamics simulations using CHARMM-GUI (https://charmm-gui.org/) [44]. Input files were prepared by embedding the proteins in bilayers composed of 1-Palmitoyl-2-oleoyl-sn-glycero-3-phosphorylcholine (POPC) lipid molecules. The complexes were placed in a triclinic box with a minimum distance of 1.0 nm between the complex atoms and the edges of the box. The system charge was neutralized using 0.15 M NaCl as the counterion. Proteins, ligands, and phospholipids were solvated in the OPC water model using AMBER19SB, GAFF2, and SLIPPER force fields to generate topology files [45,46].

Molecular dynamics simulations were performed using the GROMACS 2022.6 software package. During the dynamic simulation, the solvated system was initially energy-minimized using the steepest descent algorithm until the energy converged to 1000 kJ mol^−1^nm^−1^. Subsequently, the energy-minimized system was heated from 0–300 K over a period of 5 ns for the equilibration simulation. Positional constraints on the proteins and ligands were gradually reduced for an additional 5 ns to equilibrate the pressure in the NPT ensemble (1 bar). Following the equilibrium process, 200 ns molecular dynamics simulations were conducted at a constant temperature of 300 K and pressure of 1 bar, with a simulation time step of 2 fs. The entire process utilized the Parrinello-Rahman method to maintain the pressure and the V-rescale algorithm to maintain the temperature. Long-range electrostatic interactions were calculated using the particle mesh Ewald method, and hydrogen bonding was constrained using the LINCS algorithm. During the molecular dynamics simulation, the stability of the system was assessed by calculating the RMSD of the protein backbone [47,48].

Binding free energy calculations are commonly employed to assess the strength of the interactions between a receptor and a ligand, serving as a vital tool in biomolecular simulations. Frequently used methods for binding free energy calculations include free energy perturbation (FEP) [49] and molecular mechanics generalized boron surface area (MM-GB/PBSA) [50]. This can be expressed by the following equations:

Δ*G_bind_* = *G_complex_* − (*G_protein_* − *G_ligand_*), (3)

Δ*G* = *RT·ln K*, (4)

Δ*G_bind_* = *RT·ln K_dissociated_* = *RT·lnKd* ≈ *RT·ln*IC_50_ = −*RT*·pIC_50_, (5)

where *G_complex_*, *G_protein_*, and *G_ligand_* represent the free energies of the protein-ligand complex, the protein, and the ligand, respectively, in an implicit solvent. In this study, binding free energy calculations were performed using gmx_MMPBSA [51], based on the last 100 ns of the smooth trajectory structure obtained from the molecular dynamics simulation. The binding free energy between the hookah alkaloids and GABAAR, as well as the GlyR, was calculated along with each of the four energy terms (van der Waals, electrostatic, polar solvation, and non-polar solvation) using the MMPBSA method. Additionally, the energy contributions of the amino acids within 4 Å of the small molecule binding site were assessed, and amino acids with high energy contribution values were selected for mutation to alanine.

### 4.5. Statistical Analysis

All results are presented as mean ± SE. Statistical analyses and graph plotting were performed using Origin Pro (version 2021). Values of *p* < 0.05 was considered statistically significant. Paired or unpaired Student *t*-tests were used for statistical comparisons.

## 5. Conclusions

This study elucidated the modulatory effects of the major alkaloids of *Gelsemium* on inhibitory neuroreceptors and their mechanisms. The low-toxicity alkaloid gelsemine exerts its modulatory effects by binding to the orthosteric site of the GlyR. The GABAAR is a common target for koumine, gelsemine, gelsevirine, gelsenicine, and humantenirine, with the transmembrane region of the β+/α− interface serving as a common binding site. The present analysis of these five representative alkaloids identified their pharmacological and toxicological targets as well as their mechanisms of action. This work contributes to a broader understanding of the pharmacological and toxicological mechanisms of *Gelsemium* and provides strong support for its use in drug development.

## Figures and Tables

**Table 1:** Pharmacological actions of koumine, gelsemine, gelsevirine, gelsenicine, and humantenirine at a concentration of 300 µM on nicotinic acetylcholine receptors (nAChRs), GABAA receptors (GABAARs), and glycine receptors (GlyRs).

	Koumine	Gelsemine	Gelsevirine	Gelsenicine	Humantenirine
GABAAR	−55.54% ± 4.35%	−58.78% ± 1.74%	−55.82% ± 0.35%	58.63% ± 5.56%	41.66% ± 0.53%
α1GlyR	−95.53% ± 0.70%	−70.39% ± 4.85%	−98.89% ± 0.68%	−13.64% ± 4.34%	−11.75% ± 4.34%
α1βGlyR	−87.29% ± 0.52%	−92.18% ± 2.87%	−99.06% ± 0.62%	−10.44% ± 5.99%	−16.82% ± 4.41%
nACHR	1.42% ± 0.00%	ND	0.75% ± 0.65%	ND	ND

ND: not detected. Note the percentage change in peak current modulation of GABAAR, GlyR, and nACHR by *Gelsemium* alkaloids at a concentration of 300 µM. For GABAAR, there were no significant differences among koumine, gelsemine, gelsevirine, and gelsenicine, while humantenirine showed significant differences compared to all four alkaloids (*p* < 0.01). For α1 GlyR, koumine and gelsevirine showed significant differences compared to gelsemine, gelsenicine, and humantenirine (*p* < 0.01). For α1β GlyR, koumine, gelsemine, and gelsevirine showed significant differences compared to gelsenicine and humantenirine (*p* < 0.01). For nACHR, there was no significant difference between koumine and gelsenicine.

**Table 2:** Pharmacological actions of various concentrations of koumine, gelsemine, and gelsevirine on GlyRs and GABAARs.

	Alkaloids	IC_50_ (μM)	EC_50_ (μM)	Hillslope	n
GABAAR	Koumine	142.8	ND	−0.9283	4
	Gelsemine	170.8	ND	−0.9158	4
	Gelsevirine	251.5	ND	−1.110	4
	Gelsenicine	ND	192.1	0.8002	4
α1GlyR	Koumine	9.587	ND	1.079	4
	Gelsemine	10.36	ND	1.316	4
	Gelsevirine	82.94	ND	1.179	4

ND: Not detected.

**Table 3:** Binding free energy of the ligand-receptor complexes (in kcal/mol). Data are expressed as mean ± SE.

	Gelsemine	Gelsenicine
GABAAR Site 1	−7.44 ± 3.47	−10.88 ± 3.92
GABAAR Site 2	−10.25 ± 4.13	−14.83 ± 5.23
GABAAR Site 3	−26.89 ± 4.91	−22.75 ± 4.26
GlyR	−30.7 ± 3.42	−12.17 ± 3.85

## Data Availability

All data and materials included in this study are available upon request by contact with the corresponding author.

## Supplementary Materials

The following supporting information can be downloaded at: https://www.mdpi.com/article/10.3390/ijms26031312/s1.
