# Supplementary material for: Experimental and Computational Investigation of the Target and Mechanisms of Gelsemium Alkaloids in the Central Nervous System
Source: Int J Mol Sci. 2025 Feb 4;26(3):1312. doi: 10.3390/ijms26031312 (PMC11818404; doi:10.3390/ijms26031312)
Supplement: Supplementary file 1 [file ijms-26-01312-s001.zip › ijms-3406116-supplementary.pdf]

Supplementary Table S1 RMSD values after redocking the ligands from the crystal structures. Up to 30 of the best binding modes were retained for each system, and RMSD was calculated with reference to the ligands in the crystal structures. Results are expressed as Mean ± SD.

Binding Site	Combined model number	RMSD
Diazepam--GABAAR site1	26	0.7117±0.0537
Diazepam--GABAAR site2	30	0.6781±0.0768
Diazepam--GABAAR site3	17	0.7025±0.0583
Strychnine--GlyR	21	0.6514±0.0771

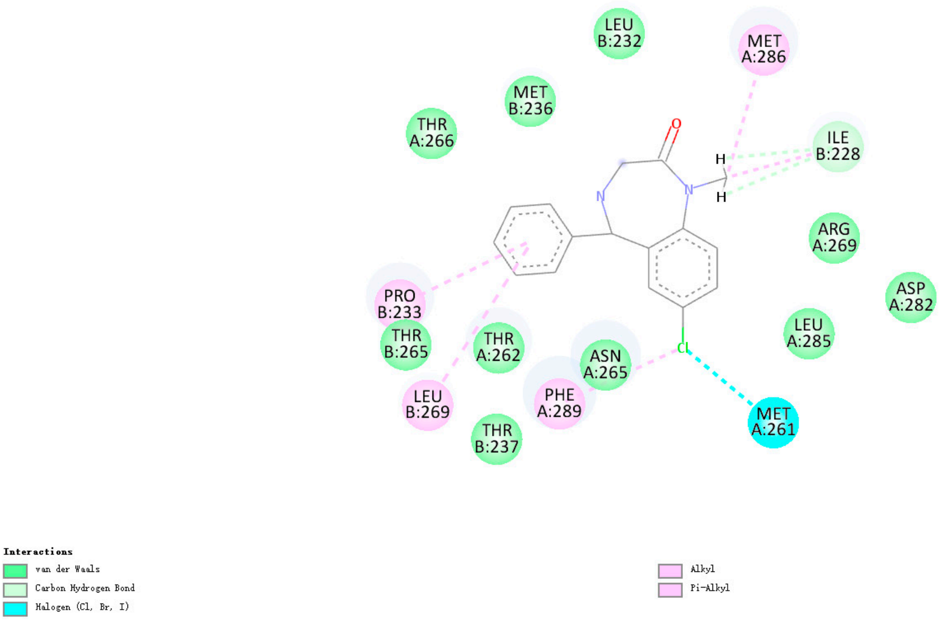

Supplementary Figure S1 Diazepam - GABAR site3 binding pattern. Diazepam forms π-alkyl interactions with PHE289 and MET286 in the β-subunit
